# HPLC-MS/MS Shows That the Cellular Uptake of All-*Trans*-Retinoic Acid under Hypoxia Is Downregulated by the Novel Active Agent 5-Methoxyleoligin

**DOI:** 10.3390/cells9092048

**Published:** 2020-09-08

**Authors:** Armin Sebastian Guntner, Christian Doppler, Christian Wechselberger, David Bernhard, Wolfgang Buchberger

**Affiliations:** 1 Institute of Analytical Chemistry, Johannes Kepler University Linz, 4040 Linz, Austria; wolfgang.buchberger@jku.at; 2 Division of Pathophysiology, Institute of Physiology and Pathophysiology, Medical Faculty, Johannes Kepler University Linz, 4020 Linz, Austria; christian.doppler@jku.at (C.D.); christian.wechselberger@jku.at (C.W.); david.bernhard@jku.at (D.B.)

**Keywords:** myocardial infarction, all-trans-retinoic acid, 5-methoxyleoligin, HPLC-MS/MS, photoisomerization

## Abstract

All-*trans*-retinoic acid (a*t*RA) is the essential derivative of vitamin A and is of interest due to its various biological key functions. As shown in the recent literature, a*t*RA also plays a role in the failing heart during myocardial infarction, the leading cause of death globally. To date insufficient mechanistic information has been available on related hypoxia-induced cell damage and reperfusion injuries. However, it has been demonstrated that a reduction in cellular a*t*RA uptake abrogates hypoxia-mediated cell and tissue damage, which may offer a new route for intervention. Consequently, in this study, the effect of the novel cardio-protective compound 5-methoxyleoligin (5ML) on cellular a*t*RA uptake was tested in human umbilical-vein endothelial cells (HUVECs). For this purpose, a high-performance liquid chromatography tandem mass spectrometry (HPLC-MS/MS) method was developed to assess intra-cellular levels of the active substance and corresponding levels of vitamin A and its derivatives, including potential *cis*/*trans* isomers. This work also focused on light-induced isomerization and the stability of biological sample material to ensure sample integrity and avoid biased conclusions. This study provides evidence of the inhibitory effect of 5ML on cellular a*t*RA uptake, a promising step toward a novel therapy for myocardial infarction.

## 1. Introduction

Vitamin A and its derivatives play major roles in numerous physiological processes, including signalling pathways; regulation of the energy metabolism; cellular proliferation and differentiation; and immune system function, growth, and development, while also maintaining health and eyesight [1,2,3,4,5,6]. Dietary uptake of vitamin A in the form of retinol or retinyl esters results in a cascade of enzymatically catalyzed modifications to retinol that yield its corresponding aldehyde and eventually retinoic acid. First, retinol dehydrogenase 10 and then retinal dehydrogenases 1–3 drive the associated oxidation reactions, the former of which is reversible [4,6,7].

Although retinol is the predominant retinoid species, its biological activity is limited. In contrast, retinoic acid (RA)—including as many as five isomers, namely all-*trans* retinoic acid (a*t*RA), 9-*cis*-RA, 13-*cis*-RA, 9,13-di-*cis*-RA, and 11-*cis*-RA—has a profound influence on body processes [8]. Further, various phase I metabolites of RA isomers, products of cytochrome P450 modification, have been reported to show biological activity [9,10,11].

According to mortality data submitted to the WHO, ischemic heart disease and its clinical manifestations consistently remain the number one cause of death, independent of income in the respective nations [12,13]. Here, immediate medical treatment including lysis techniques and mechanical circumvention (stents, bypasses) enable a direct response to a patient’s life-threatening situation [14]. The pathophysiological mechanism in the failing heart seems to offer an additional opportunity for intervention and a possible approach to lowering the mortality rate [15]. In this context, studies on rodents have shown that retinoic acid signalling is induced in the heart upon hypoxia, resulting from a myocardial infarction [2]. Further, a*t*RA levels have been shown to rise in the hearts ofaffected humans and accumulate in failing organs, while downregulation of cellular a*t*RA uptake prevents cell and animal death under hypoxia [15,16]. The cellular uptake of retinoids, however, is not fully understood; it has been suggested that retinol is taken up and transformed locally to form a*t*RA, but direct uptake of a*t*RA has also been considered [15]. It is also known that, in the human body, retinol and its derivatives are transported by the retinol-binding protein and the transmembrane transport receptor STRA6, which facilitates cellular uptake [7,15].

A recent study conducted in this field by Danzl et al. [15] has shown inhibition of a*t*RA uptake by 5′-methoxyleoligin (5ML) in cell culture experiments. The in vitro experimental setup was designed to mimic a post-myocardial infarct situation and showed a decrease in hypoxia-induced cell death in cultures with 5ML present [15]. While cell death can be ascertained directly by the annexin V/propidium iodide method and lactate dehydrogenase release assays, previous studies determined a*t*RA only by indirect measurement using, for instance, RARE-Luciferase reporter gene assays [15].

**Figure 1:**
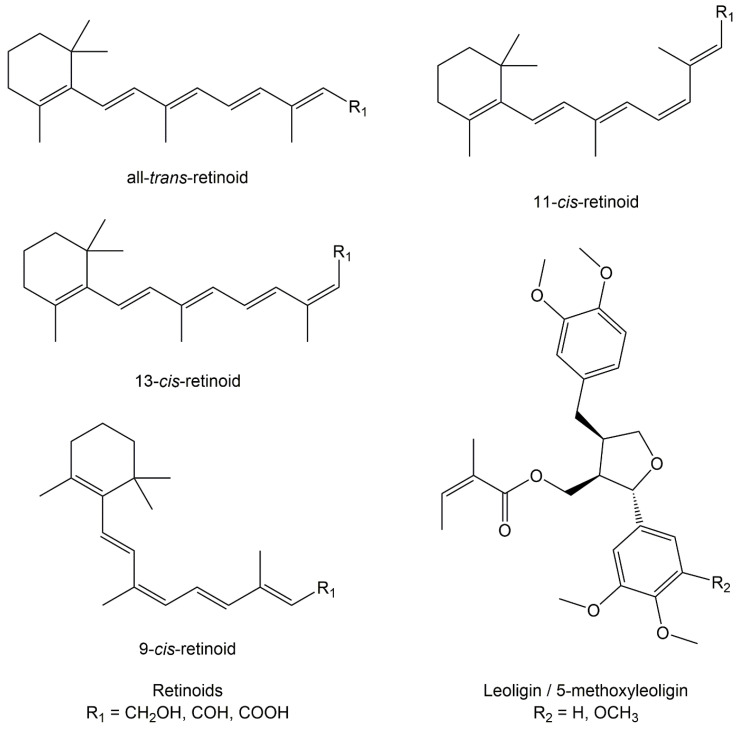
Overview of the substances under investigation.

To improve data quality by direct determination of analytes for the purpose of further drug development and optimization, one main aim of this study was to introduce a new sensitive and selective high-performance liquid chromatography tandem mass spectrometry (HPLC-MS/MS) method for quantitative determination of intracellular retinoid and 5ML levels. Various methods using LC-MS systems to analyze retinoids have been published [17,18,19,20,21], but—to the best of our knowledge—none of them covered all target analytes. Accordingly, it was our goal to extend the analysis to include more retinoid compounds (see Figure 1) and to achieve a more comprehensive understanding of retinoids and their metabolisms. Further, published methods were not capable of separating leoligin and its derivatives together with retinoids within one run. For the present application, detection limits in the low microgram per liter range were required, as well as chromatographic conditions allowing a reliable separation of isomers that cannot be differentiated by MS detection. In this context, Figure 1 provides an overview of the investigated compounds in the present study. 

Finally, note that the validity of bioanalytical assays relies heavily on the integrity of the sample material [22]. This is particularly relevant to retinoid analytics due to their known light sensitivity and a possible induction of *cis*/*trans* isomerization [23,24,25,26], which is also known to be central to the process of vision [27]. Consequently, we investigated related reactions from an analytical point of view to avoid biased isomer quantitation results. More specifically, an in-house developed in situ isomerization approach was used to test for sample integrity upon sample preparation.

## 2. Materials and Methods

Retinol (≥95%), a*t*RA (≥98%), 9-*cis*-RA (≥98%), and 13-*cis*-RA (≥98%) all-*trans* retinoic aldehyde (a*t*RAL) (≥98%) were purchased from Sigma-Aldrich Handels GmbH (Vienna, Austria). Stock solutions were prepared in Dimethyl sulfoxide (DMSO), further diluted as needed using methanol, and stored in the dark at 4 °C. Leoligin and 5ML from *Leontopodium alpinum*, obtained as described in Schwaiger et al. [28], were acquired from the Institute of Pharmacy/Pharmacognosy, Leopold-Franzens University Innsbruck, Innsbruck, Austria. Purity (>98%) was ensured using nuclear magnetic resonance spectroscopy. All solvents used were HPLC grade and were purchased from VWR International GmbH (Darmstadt, Germany). Formic acid (≥96%) was purchased from Sigma-Aldrich (Vienna, Austria). Water was used in Millipore quality obtained from a Millipore purification system (Molsheim, France).

Primary human umbilical vein endothelial cells (HUVECs) were purchased from Promocell (Vienna, Austria). For the trials, 400,000 cells were seeded per 6 cm dish and incubated overnight at 37 °C and 5% CO_2_. For analyzing the time-depended uptake of a*t*RA and retinol under hypoxia, cell culture medium was replaced with fresh medium that contained growth factors but no serum, and cells were treated with either 10 µM a*t*RA, or 10 µM retinol, or DMSO as solvent control. Working concentrations of a*t*RA and retinol were chosen in accordance to the protocols described by Danzl et al. [15]. The dishes were transferred to hypoxia chambers (Billups-Rothenberg, Inc., San Diego, CA, USA), which were then flushed with nitrogen gas for 5 min at 2 L min^−1^ to reduce the atmospheric oxygen level to 0.3%. Following incubation periods of 15 min, 30 min, 60 min, 120 min and 360 min at 37 °C, as described by Danzl et al. [15], and prior to harvesting, cells were washed twice with PBS to remove all extracellular a*t*RA or retinol. Subsequently, cells were trypsinized and centrifuged. The resulting cell pellets were lysed by addition of acetonitrile/ethanol/water (2:2:1) followed by 2 min of ultrasonic treatment. The supernatant was collected after centrifugation for 10 min at 500 rcf and used for further analysis. All analyses were performed in triplicate and under light protection. For investigating the influence of 5ML treatment on cellular retinoid uptake under hypoxia, cell culture medium was replaced with fresh medium containing growth factors but no serum, and cells were treated with either 10 µM a*t*RA, or 10 µM retinol, or 30 µM 5ML or a combination thereof. Then, 5ML was added to the cells 12 h before (time point –12 h) or concurrently with (time point 0 h) induction of hypoxia, and DMSO was used as solvent control. Cells were washed twice with PBS to remove remaining a*t*RA, retinol or 5ML and harvested after incubation at 37 °C for 15 min or 360 min. Concentrations and incubation times were set in agreement with [15]. Cell lysis and subsequent sample preparation steps were conducted as stated above. All statistical analyses of cellular experiments were performed with SPSS statistics 22 software (IBM, Armonk, NY, USA). Data was tested for Gaussian distribution by the Shapiro Wilk test and further analyzed using two-sided t-test comparisons. Groups were defined to differ significantly at a *p* value < 0.05; all analyses were performed in triplicates.

For chromatographic separation of analytes and matrix constituents, a 1260 Infinity Series HPLC combined with a guarded C18 Poroshell 120 column (150 × 3 mm; 2.7 µm; endcapped silica), both from Agilent Technologies (Santa Clara, CA, USA) was used. Quantitation was achieved with a 6420 triple quadrupole mass spectrometer (QqQ MS) from Agilent Technologies (Santa Clara, CA, USA). For determining collision cross sections, the HPLC system described above was coupled to a 6560 ion-mobility quadrupole time-of-flight MS (IMS-Q-TOF MS) from Agilent Technologies (Santa Clara, CA, USA). MassHunter Workstation LC/MS Data Acquisition 10.0 SR1 software (Agilent Technologies, Santa Clara, CA, USA) was employed for setup control and acquisition procedures. Automated data processing was achieved using MassHunter Workstation Quantitative Analysis for QQQ 10.0 SR1 software (Agilent Technologies, Santa Clara, CA, USA). Precursor ion fragmentation in MS/MS experiments and electrospray ionization efficiency were optimized with MassHunter Workstation Optimizer and Source Optimizer 10.0 SR1 software (Agilent Technologies, Santa Clara, CA, USA) to ensure sensitivity.

To enable chromatographic separation of analyte from matrix constituents and to ensure sensitivity of the assay, multiple system parameters were optimized during method development. Previously published assays [19,29] served as a starting point, but were adjusted considerably to meet our requirements. Target analytes were separated within 30 min (including a 5 min column-reconditioning phase) at a flow rate of 0.5 mL min^−1^, a column compartment temperature of 45 °C, and a gradient of water (A) and acetonitrile (B)—both modified with 0.1 v% formic acid. The initial proportion of 55 v% A was decreased to 40 v% within 10 min and further decreased to 28 v% at 15 min. The reduction of the aqueous fraction was continued to 1 v% at 24 min and the composition of the mobile phase was kept constant for 1 min. At 25 min, the eluent mixture was returned to its initial proportions for reconditioning. In all analyses, mass spectrometry was used for detection, for which a multiple-reaction monitoring method was developed to ensure selectivity for and sensitivity to the target analytes.

As ionization efficiency is key in determining both sensitivity and accuracy in HPLC-MS/MS analysis that is heavily influenced by matrix co-elution, in situ matrix-matched calibration was used to compensate for possible matrix-induced suppression/enhancement effects. An automated sampler program was therefore employed to inject the same amounts of pooled matrix into the system for calibration standards as for sample analysis. In detail, the 1260 Infinity II series HPLC autosampler was set to draw 5 µL of matrix pool and 5 µL of standard solution and mix the resulting 10 µL in the injector needle 5 times prior to injection. In the case of sample analysis, 5 µL was thus injected. Additionally, time segments were used, to control the actual HPLC flow to the MS in order to avoid excessive matrix contamination of the ionization source and the overall system. For two periods of the 30 min chromatographic run—9 min to 15 min and 20 min to 26 min—the solvent flow was directed to the ionization source; the remainder was discarded. Electrospray ionization parameters were set in accordance with results of the optimization procedure to increase signal intensities and thus sensitivity of the assay. For proper ionization, the drying gas was set to a temperature of 350 °C and a flow rate of 12 L min^−1^. The nebulizer pressure was adjusted to 60 psi. Further, a capillary voltage of 4 kV was used in positive polarization. MS/MS detection is known to allow both high sensitivity and high selectivity and was consequently employed in all quantitative analyses presented in this study. Based on results of initial optimization experiments using MassHunter Workstation Optimizer 10.0 SR1 software, the most suitable MS/MS parameters were chosen, as listed in Table 1. All measurements were conducted at 7 V cell accelerator voltage and at 500 V electron multiplier voltage.

In addition to chromatographic separation of isomers with MS/MS detection, we tested ion mobility mass spectrometry (IMS-MS) for its ability to achieve the required isomer selectivity. In principle, this technique enables separation of isomeric compounds within milliseconds thus faster chromatographic runs because isomer separation may be achieved in the coupled IMS-MS and may therefore not be necessary in the LC system [30].

Photoisomerization experiments were carried out using an InGaN surface-mounted LED (NPW-TSD-ST-1) with a luminous flux of 51.7–87.4 lm (depending on the viewing angle) at 3.5 V, purchased from Dominant Semiconductors Europe GmbH (Bad Rappenau, Germany). The LED was operated with an adjustable power supply (Voltcraft SNG-1000-OC power supply, Conrad Electronic AG, Wollerau, Suisse) for setting light intensity. These experiments were conducted to test for light-induced degradation/transformation of target analytes that might lead to biased quantitation results of cell culture samples. For this purpose, a surface-mounted InGaN LED was attached to an Agilent 1100 series autosampler vial plate and installed in an Agilent 1260 autosampler (Agilent Technologies, Santa Clara, CA, USA). These system components were relevant here, because the vial plate used had openings beneath the samples that allowed a constant illumination during the photoisomerization experiment. Figure S1 of the supplementary material provides a schematic representation of the workflow for isomer generation and online analysis. The corresponding experiments were carried out in typical sample vials with either clear or amber glass, using various illumination intensities. In detail, 1.5 mL of an acetonitrile/water solution (75/25) containing 1 mg L^−1^ retinol and a*t*RA was illuminated, sampled automatically at certain time points using the setup described above, and analyzed by means of the optimized HPLC-MS/MS method for detecting retinoids and leoligins. The presented approach allowed time-dependent in situ generation of isomers and subsequent online analysis. The sample rate was limited by chromatography to two data points per hour but was increased at the beginning of each experiment, as preliminary tests had shown a more pronounced change of the retinoid isomer profile within the first 60 min of illumination. To cope with this, an autosampler program was set to transfer defined amounts of test solution into shielded storage vials with micro inserts every 10 min. These solutions were measured after the illumination experiments. Due to the high thermal stability of *cis* and *trans* isomers [31], the storage at low temperatures largely avoids systematic errors. All photoisomerization experiments were ran for 24 h at the described sample rate (maximum of two data points per hour), with the exception of laboratory light exposure experiments, where the sample rate was considerably lower due to manual sampling. In these tests, illumination was achieved using typical laboratory light of ceiling-mounted fluorescent tubes.

## 3. Results and Discussion

### 3.1. HPLC-MS/MS and HPLC-IMS/MS Analysis of Retinoid and Leoligin Species

**Figure 2:**
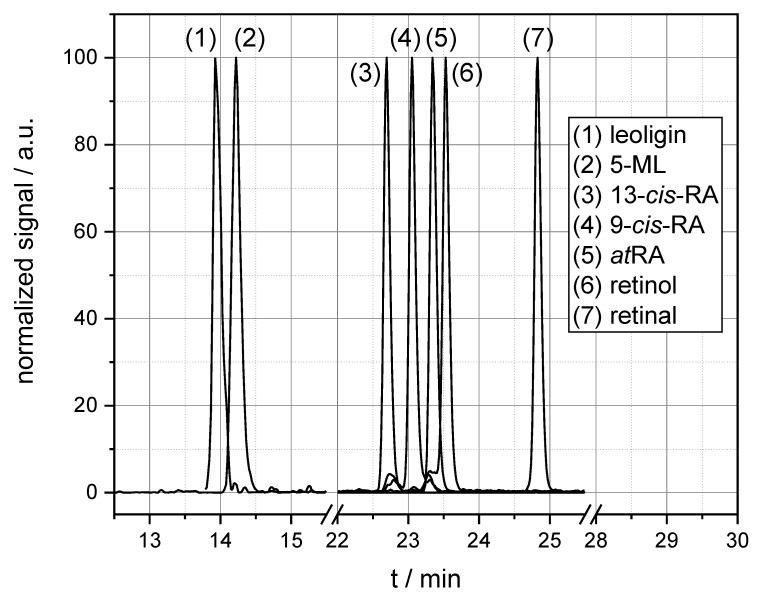
Normalized chromatogram obtained with the high-performance liquid chromatography tandem mass spectrometry (HPLC-MS/MS) method developed.

Based on the HPLC-MS/MS parameter optimization described in the Materials and Methods section, the target analytes were separated and detected with sufficient selectivity and sensitivity. For this purpose, a bespoke chromatographic gradient was developed that ensured overall method performance. Focusing on precise retention times in the HPLC analysis ensured isomer selectivity, whilst the high sensitivity of the hyphenated QqQ MS allowed intracellular quantitation, which was the primary aim of the study. The separation of retinoid isomers was essential, as it had to be determined whether a possible decrease of intracellular a*t*RA levels originated from the treatment with the active substance or an unintentional light-exposure. Moreover, it was important for the quantitative analysis to confirm initially that mass spectrometric signals were clearly assignable to the target compounds and not distorted by underlying isomer signals. Figure 2 provides a typical chromatogram obtained with the method developed. For all analytes, the method was linear between the limit of detection and 1 mg L^−1^. Method standard deviation, reflecting precision, was below 10%, and the limit of quantification was below 4 µg L^−1^ for all target analytes.

Besides, ion mobility mass spectrometry analysis of retinoids was additionally conducted to test for a possible drift time separation of isomers and to ascertain the projection of the three-dimensional structure of the analytes. We were able to determine collision cross sections but could not separate isomers in the IMS-MS experiments, which may be attributed to reactions occurring during the electrospray ionization process. Previous ion mobility studies have described the formation of equal amounts of *trans* and *cis* isomers of protonated Schiff base retinal after electrospraying, concluding that the energy barriers for conversion are comparably low (lower than for those for thermal isomerization) [32]. It has been shown that a*t*RA anions, in contrast, preserve their isomerization profile upon electrospraying, but undergo ultrafast isomerization when exposed to pulsed laser light in the gas phase [31]. Note that—as for the protonated Schiff base retinal studied in [32]—for protonated retinoic acid multiple resonance structures are present because the positive charge is delocalized along the carbon chain of the molecule (cf. Figure 1). This, in turn, results in a bonding character somewhere between double and single bond that allows rotation and subsequent isomerization [32]. In addition, comparing calculated collision cross sections to those measured by IMS-MS analysis suggests that electrospray ionization may be followed by a cyclization reaction in a Diels-Alder-like mechanism [33]. In detail, IMoS CCS calculations, as described by Guntner et al. [34], predicted the following CCS: 9-*cis*-RA: 192.93 Å^2^; 11-*cis*-RA: 194.76 Å^2^; 13-*cis*-RA: 199.27 Å^2^; a*t*RA: 203.09 Å^2^. The CCS of RA measured in our IMS-MS analysis was 176.39 Å^2^, which reflects a much more compact structure in the gas phase than expected from the native structure. Separation of retinol isomers by ion mobility was not possible, because *all*-*trans*-retinol and the corresponding *cis* isomers exhibited the same drift times. This, however, may be related to the fact that electro-sprayed retinol loses water due to in-source fragmentation and eventually forms a charged, dehydrated species. Again, multiple resonance structures can be assumed, as the positive charge may be delocalized along the carbon chain of the molecule. 

### 3.2. Photoisomerization Analysis of Retinol and Retinoic Acid

Photoisomerization experiments with retinol and a*t*RA were conducted to test for storage stability and to estimate possible systematic errors introduced into the assay by prolonged sample preparation times under illumination. As described in the Materials and Methods section, an in situ approach generating isomers in solution in combination with HPLC-MS/MS analysis was used in this context. Multiple experiments using either the surface-mounted LED described above at various illumination intensities or laboratory illumination (fluorescent ceiling lights) were conducted in clear glass or amber vials. In general, as given in Equations (1)–(3), light exposure may transform isomer *A* into isomer *B* with a particular quantum yield and within a particular time t, in accordance with general kinetics and corresponding thermal (K) and photochemical (J) rate constants [35]:

(1) $$A\;\overset{hv}{\to }B$$

(2) $$-\frac{d\left[A\right]}{dt}=\;\frac{d\left[B\right]}{dt}=\left(J_{A\to B}+K_{A\to B}\right)\left[A\right]-K_{B\to A}\left[B\right]$$

Since photochemical rate constants are proportional to the total light intensity, Equation (2) can be simplified to [35]

(3) $$\left[A\right]=Pe^{R_{0}t}+Q$$

where $R_{0}$ is the sum of all rate constants, Q is the asymptotic concentration of an isomer in the photostationary state, and P represents the difference of initial and asymptotic isomer concentration [35].

**Figure 3:**
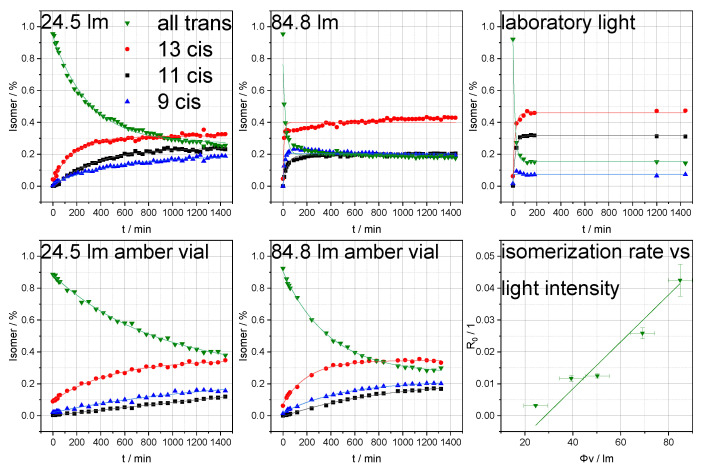
Isomer profiles of retinoic acid at various light intensities and in two different vials, measured at various time points. Additionally, the isomerization rate as a function of light intensity is displayed (error bars represent deviations in light intensity of the LED and isomerization rate).

**Figure 4:**
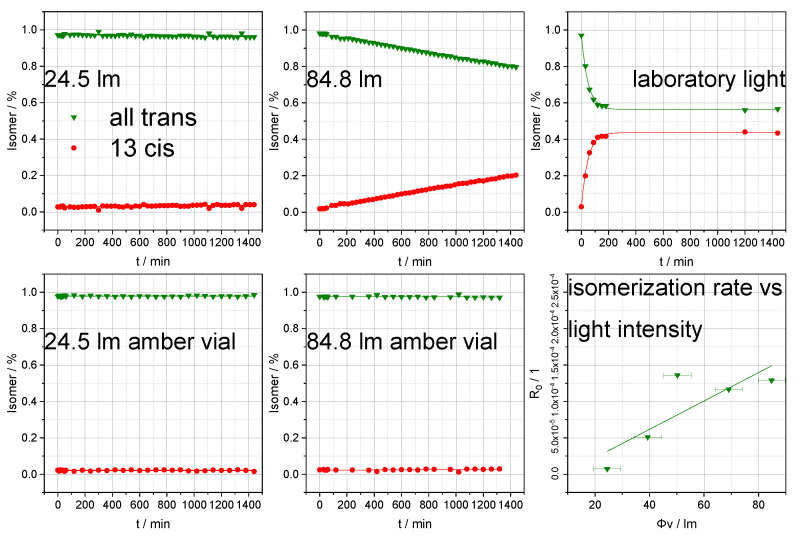
Isomer profiles of retinol at various light intensities and in two different vials, measured at various time points. Additionally, the isomerization rate as a function of light intensity is displayed (error bars represent deviations in light intensity of the LED and isomerization rate).

Figure 3 and Figure 4 show that illumination of a retinoid sample solution immediately leads to the formation of isomers. The corresponding reaction speeds are thereby proportional to the light intensities and were tested using five distinct illumination intensities (24.5 lm, 39.3 lm, 50.3 lm, 69.0 lm, and 84.8 lm). A comparison of the results for the reactions under LED and laboratory light conditions shows that the decrease in isomer concentration while approaching the photostationary state depends both upon irradiation intensity and upon the wavelength distribution of the illumination source [35]. Normalized illumination intensities of the exposed area were significantly higher using the LED than under laboratory light conditions [36]. However, the corresponding experiments showed comparable or even more pronounced isomerization rates for laboratory light, most probably due to commercial fluorescent tubes emitting a small proportion of UV light close to the absorption maxima of retinoids [37,38]. Under the present conditions, retinoic acid responded more strongly than retinol to illumination. Considering the generally fast response of the retinoid isomer profile to irradiation, reducing illumination to a minimum is crucial for obtaining unbiased quantitation results.

### 3.3. Results of Targeted Retinoid Analysis in HUVEC Experiments Including 5ML Treatment

**Figure 5:**
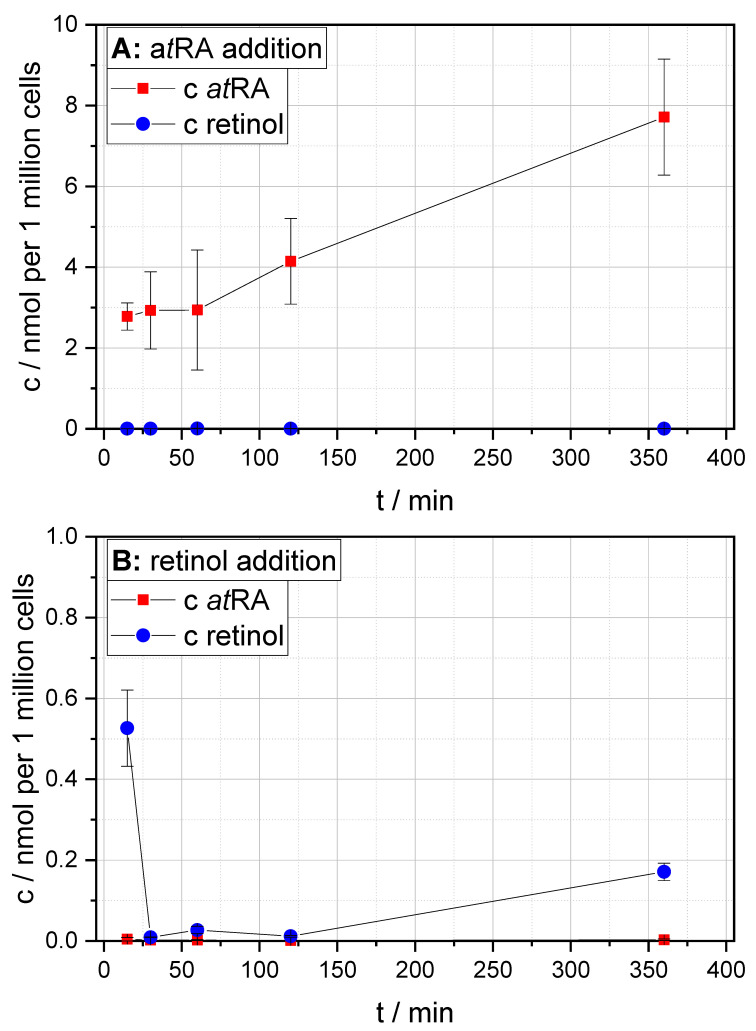
Results of the in vitro tests measuring the uptakes of all-*trans* retinoic acid (a*t*RA) (**A**) and retinol (**B**) by human umbilical-vein endothelial cells (HUVECs) under hypoxic conditions at various time points. The error bars represent the standard deviation of the results of biological triplicates.

In the first step, to test for general cellular uptake of retinoids, HUVECs were kept under hypoxic conditions and treated with a*t*RA or retinol or DMSO as solvent control. After cell lysis and sample preparation, HPLC-MS/MS was used for quantification. Figure 5 provides detailed insights into the time-dependent intracellular quantitation results for a*t*RA (Figure 5A) and retinol (Figure 5B) uptakes under hypoxia. As can be seen, the uptake of a*t*RA under hypoxic conditions was significant, and intracellular levels rose throughout the experiment. The retinol uptake was less pronounced. Further, the intracellular retinol concentrations dropped immediately after an initial peak and then rose gradually until the end of the experiment. Further, comparison with the intracellular levels of a*t*RA and retinol in cells treated with the DMSO solvent control (data not shown, as a*t*RA and retinol levels were constantly below the assay’s LOQ) clearly demonstrates the significant uptake of these compounds.

**Figure 6:**
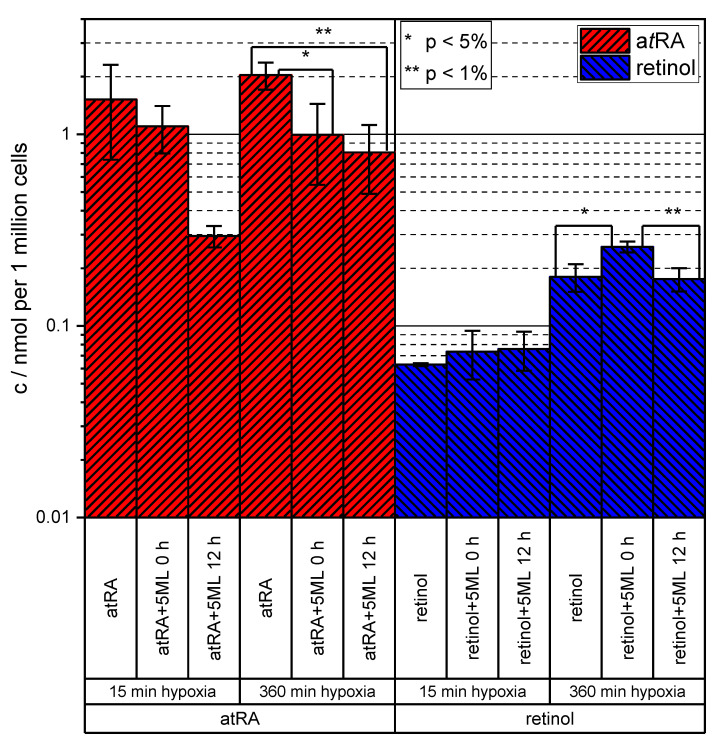
Results of the in vitro experiment measuring the inhibitory effect of 5ML on the a*t*RA uptake of HUVECs under hypoxic conditions. Hypoxic conditions were maintained for 15 min and for 360 min. Here, 5ML was added 12 h before the start of hypoxia or concurrently (0 h). The error bars represent the standard deviations of biological triplicates.

Subsequently, the influence of 5ML treatment on the uptakes of a*t*RA and retinol by HUVECs under hypoxia was tested quantitatively (a drop in intracellular a*t*RA levels under hypoxic conditions due to 5ML treatment had already shown qualitatively [15]). To this end, HUVECs were kept under hypoxia and treated with either a*t*RA or retinol or DMSO as control. Then, 5ML was added either 12 h before or concurrently with the induction of hypoxia. As can be seen in Figure 6, 5ML treatment reduced the uptake of a*t*RA in the 15 min experiments. The effect was stronger when 5ML was added 12 h before induction of hypoxia (a*t*RA 15 min/a*t*RA 15 min + 5ML 12 h: *p* = 0.056). For 360 min of hypoxia, both administration of 5ML 12 h in advance and at induction led to a significant decrease of cellular a*t*RA uptake (a*t*RA 360 min/a*t*RA 360 min + 5ML 0 h: *p* = 0.031, a*t*RA 360 min/a*t*RA 360 min + 5ML 12 h: *p* = 0.009). For retinol, no such inhibitory effect of a 5ML treatment was observable in the 15 min experiments. Instead, under hypoxic conditions for 360 min, more retinol (retinol 360 min/retinol 360 min + 5ML 0 h: *p* = 0.017) was taken up by the cells when 5ML was applied at induction. When 5ML was added before induction, the retinol levels were the same as observed for the control, but the difference to the concurrent addition was significant (retinol 360 min + 5ML 0 h/retinol 360 min + 5ML 12 h: *p* = 0.009).

## 4. Conclusions

In this study, the effect of 5ML on cellular a*t*RA uptake under hypoxic conditions was quantitated using HPLC-MS/MS. The results represent a significant step forward in the development of a new potential drug for the treatment of myocardial infarction. We have verified published qualitative data, which aligns well with the results presented here. In addition, time-dependent cellular uptake of retinoids under hypoxic conditions was measured showing a significant increase in cellular a*t*RA levels.

In developing the bioanalytical assay, we focused on general sensitivity and selectivity, and particularly on retinoid isomer separation, to assure the quality of the resulting quantitative data. Inadequate separation and thus insufficient selectivity for the target compounds would lead to less reliable quantitative results, since mass spectrometry alone cannot make a distinction here. Our test of the suitability of ion-mobility analysis for separating isomers revealed that this technique is not suitable, most probably due to reactions occurring upon electrospraying.

Strong emphasis was put on sample integrity to ensure unbiased results, especially because retinoid compounds are highly light-sensitive. In an extensive study of photoisomerization and light sensitivity, we found that exposure to light is an immediate source of error throughout all steps of the bioanalytical assay: from sampling to sample preparation and to the actual HPLC-MS/MS analysis, there is the potential systematic risk of isomerization causing changes in the profile of the retinoids present, which inevitably leads to erroneous conclusions.

Finally, a key and highly relevant result of this study is that addition of a*t*RA to cells in culture leads to increased intracellular levels of a*t*RA, which strongly supports the view that there may be a direct, active a*t*RA uptake that is susceptible to downregulation by 5ML. The possibility of direct interaction between leoligins and retinoids clearly needs to be tested.

Independent of its mode, inhibition of cellular retinoid uptake by leoligins offers great potential for the treatment of a variety of diseases, including the number one killer worldwide—myocardial infarction.

## Figures and Tables

**Table 1:** Parameters and mass transitions for tandem mass spectrometry based quantitation of leoligins and retinoids.

Compound	Precursor Ion m/z	Product Ion m/z	Dwell Time/ms	Fragmentor Voltage/V	Collision Energy/eV
5ML	523.33	423.2	150	106	18
		181.1			34
		151.1			50
leoligin	493.2	393.2	150	162	14
		151.0			22
		107.0			90
a*t*RA	301.22	205.0	150	76	10
		123.1			18
		91.0			58
retinal	285.2	161.1	250	102	10
		105.1			34
		91.1			58
retinol	269.3	119.2	150	114	18
		93.0			26
		78.9			26

## Supplementary Materials

The following are available online at https://www.mdpi.com/2073-4409/9/9/2048/s1, Figure S1. Scheme of in situ photoisomer generation and online HPLC-MS/MS analysis.
