# Supplementary figures and images for: HPLC-MS/MS Shows That the Cellular Uptake of All-Trans-Retinoic Acid under Hypoxia Is Downregulated by the Novel Active Agent 5-Methoxyleoligin
Source: Cells. 2020 Sep 8;9(9):2048. doi: 10.3390/cells9092048 (PMC7563598; doi:10.3390/cells9092048)

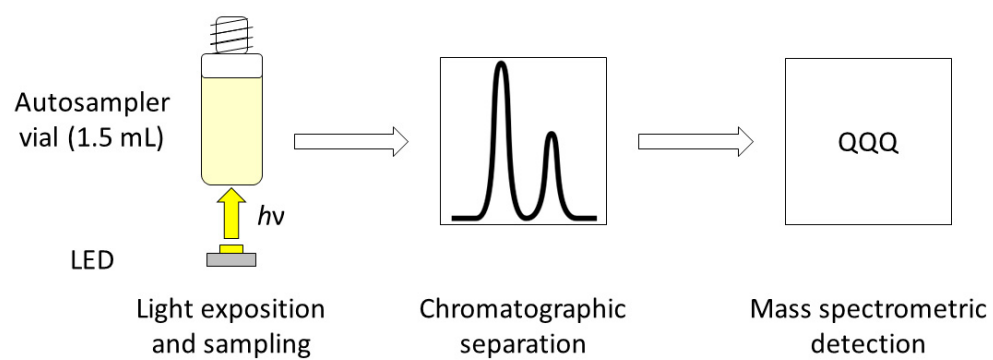

Supplement: Supplementary file 1 [file cells-09-02048-s001.pdf]
